# Controlled Reperfusion Strategies Improve Cardiac Hemodynamic Recovery after Warm Global Ischemia in an Isolated, Working Rat Heart Model of Donation after Circulatory Death (DCD)

**DOI:** 10.3389/fphys.2016.00543

**Published:** 2016-11-22

**Authors:** Emilie Farine, Petra Niederberger, Rahel K. Wyss, Natalia Méndez-Carmona, Brigitta Gahl, Georg M. Fiedler, Thierry P. Carrel, Hendrik T. Tevaearai Stahel, Sarah L. Longnus

**Affiliations:** ^1^Clinic for Cardiovascular Surgery, Inselspital, Bern University HospitalBern, Switzerland; ^2^Department of Clinical Research, University of BernBern, Switzerland; ^3^Center of Laboratory Medicine, University Institute of Clinical Chemistry, University HospitalInselspital, Bern, Switzerland

**Keywords:** acidic reperfusion, cardioprotection, donation after circulatory death, heart transplantation, hypoxia, ischemia reperfusion injury, mechanical postconditioning, mild hypothermia

## Abstract

**Aims:** Donation after circulatory death (DCD) could improve cardiac graft availability, which is currently insufficient to meet transplant demand. However, DCD organs undergo an inevitable period of warm ischemia and most cardioprotective approaches can only be applied at reperfusion (procurement) for ethical reasons. We investigated whether modifying physical conditions at reperfusion, using four different strategies, effectively improves hemodynamic recovery after warm ischemia.

**Methods and Results:** Isolated hearts of male Wistar rats were perfused in working-mode for 20 min, subjected to 27 min global ischemia (37°C), and 60 min reperfusion (n = 43). Mild hypothermia (30°C, 10 min), mechanical postconditioning (MPC; 2x 30 s reperfusion/30 s ischemia), hypoxia (no O_2_, 2 min), or low pH (pH 6.8–7.4, 3 min) was applied at reperfusion and compared with controls (i.e., no strategy). After 60 min reperfusion, recovery of left ventricular work (developed pressure^*^heart rate; expressed as percent of pre-ischemic value) was significantly greater for mild hypothermia (62 ± 7%), MPC (65 ± 8%) and hypoxia (61 ± 11%; p < 0.05 for all), but not for low pH (45 ± 13%), vs. controls (44 ± 7%). Increased hemodynamic recovery was associated with greater oxygen consumption (mild hypothermia, MPC) and coronary perfusion (mild hypothermia, MPC, hypoxia), and with reduced markers of necrosis (mild hypothermia, MPC, hypoxia) and mitochondrial damage (mild hypothermia, hypoxia).

**Conclusions:** Brief modifications in physical conditions at reperfusion, such as hypothermia, mechanical postconditioning, and hypoxia, improve post-ischemic hemodynamic function in our model of DCD. Cardioprotective reperfusion strategies applied at graft procurement could improve DCD graft recovery and limit further injury; however, optimal clinical approaches remain to be characterized.

## Introduction

Unfortunately, insufficient supply of cardiac grafts has become an obstacle in heart transplantation. Importantly, heart transplantation remains the gold standard treatment for improving quality of life and survival in severe cases of heart failure, one of the leading causes of morbidity and mortality in western countries. In Europe, 6438 patients were registered on waiting lists for heart transplantation in 2014 and 567 patients (~8.8%) (Council of Europe Matesanz, 2015) died while awaiting a donor heart. Increasing organ availability with donation after circulatory death (DCD) (Longnus et al., 2014), in addition to conventional donation after brain death, is one promising option to overcome the organ shortage.

Although the heart transplantation was first performed with DCD (Barnard, 1967), DCD has generally not been considered for cardiac donation due to concerns about irreversible cardiac injury arising from warm ischemia. Unlike donation after brain death, DCD organs undergo an inevitable period of warm, unprotected, global ischemia between circulatory arrest and graft procurement. Recent reports of successful DCD heart transplantations in Australia (Dhital et al., 2015) and in the UK (Leprince et al., 2016; Macdonald et al., 2016; Page et al., 2016) attest to the feasibility of DCD heart transplantation and the ability of hearts to withstand a limited period of warm ischemia (20–30 min) (Iyer et al., 2014). Nonetheless, in order to make DCD heart transplantation a routine approach, clinical strategies that limit reperfusion injury and optimize post-transplant graft function remain to be characterized.

With DCD, organ protection strategies applied at the time of reperfusion hold particular promise, given that protective treatment options are limited before and during ischemia for ethical reasons. As reperfusion injury contributes to up to 50% total myocardial damage (Yellon and Hausenloy, 2007; Hausenloy and Yellon, 2013), approaches to limit this injury could provide an important clinical impact. Reperfusion injury is mediated, in the immediate minutes after ischemia, by increased calcium overload, rapid correction of pH, and mitochondrial reenergization, which causes a burst in reactive oxygen species production (Yellon and Hausenloy, 2007). These processes converge to induce cardiomyocyte hypercontracture and/or to promote the opening of the mitochondrial permeability transition pore (mPTP), which may ultimately lead to cardiomyocyte death. Inhibiting or slowing these processes may therefore improve graft recovery.

The conditions present in DCD heart transplantation may be particularly amenable to cardioprotective reperfusion strategies. Identification of robust cardioprotective reperfusion strategies has been the subject of intense research activity for several years, but promising pre-clinical approaches have seldom provided convincing cardioprotection in patients with ST-segment elevation myocardial infarction (STEMI) (Downey and Cohen, 2009; Lloyd-Jones, 2010; Kloner, 2013; Bulluck et al., 2016). This might be explained by interfering effects of co-morbidities and/or concomitant therapies (Hausenloy and Yellon, 2004; Bell and Yellon, 2012), delayed application of cardioprotective strategies (Roubille et al., 2014) and/or too long or too short ischemia (Barsukevich et al., 2015). However, with DCD cardiac grafts, donors will be subject to donor selection, and thus patients will generally be young and healthy, thereby limiting possible negative effects associated with comorbidities and medical therapies. Furthermore, timely application of strategies should be achievable as the duration of ischemia will be limited in DCD settings.

Several reperfusion strategies have the potential to improve post-ischemic hemodynamic function in the setting of DCD. For example, hypoxic and acidic reperfusion would be expected to slow the production of reactive oxygen species and the correction of intracellular pH, respectively; thereby reducing reperfusion injury (Serviddio et al., 2005; Angelos et al., 2006; Inserte et al., 2008; Duan et al., 2011). It is also well recognized that decreasing temperature during ischemia and/or reperfusion leads to a better recovery (Mochizuki et al., 2012; Stadelmann et al., 2013). Finally, mechanical postconditioning (MPC), brief, intermittent periods of ischemia applied at the onset of reperfusion, has also been demonstrated as cardioprotective (Ferrera et al., 2015; Bartkevics et al., 2016). However, the majority of studies performed to evaluate reperfusion strategies have used models of regional ischemia induced by temporary occlusion of a coronary artery, with analysis of the infarcted area as the typical endpoint (Rossello and Yellon, 2016), and the contractile function of the heart is rarely assessed. As such, findings from these studies cannot be easily extrapolated to situations with clinically relevant DCD conditions, including high pre-ischemic fatty acids levels, warm, global ischemia and hemodynamic evaluation.

Therefore, we hypothesized that reperfusion strategies, applied immediately following ischemia, such as mild hypothermia, mechanical postconditioning (MPC), hypoxia and mildly acidic reperfusion, could improve hemodynamic recovery after warm, global ischemia in our isolated working rat heart model of DCD.

## Materials and methods

### Experimental design

The objective of this experimental study was to test the hypothesis that the application of reperfusion strategies effectively improves post-ischemic hemodynamic function in an isolated rat heart model of DCD using a prospective, 5-arm parallel, randomized controlled design.

### Materials

Bovine serum albumin (fatty-acid free) and palmitate were obtained from Sigma–Aldrich (Buchs, Switzerland). All other chemicals were acquired from Merck (Darmstadt, Germany).

### Ethics statement

All experimental procedures were performed in compliance with the European Convention for Animal Care and approved by the Swiss animal welfare authorities and state veterinary office (Ethics Committee for Animal Experimentation (ECAE), Berne, Switzerland). Surgery was performed under anesthesia and all efforts were made to minimize animal suffering.

### Isolated heart preparation

Isolated, working rat hearts were prepared as previously described (Dornbierer et al., 2012). Adult male Wistar rats (*n* = 43; 400 ± 24 g; Janvier labs, Le Genest-Saint-Isle, France), housed under standard conditions with unlimited access to food (standard laboratory diet *ad libitum*) and water, were anesthetized intraperitoneally using 100 mg/kg of ketamine (Narketan®, Vetoquinol AG, Berne, Switzerland) and 10 mg/kg of xylazine (Xylapan®, Vetoquinol AG, Berne, Switzerland). Ketamine and xylazine may interfere with the sympathetic nervous system (Juang et al., 1980; Svorc et al., 2013), which could potentially play a confounding role in our model of ischemia and reperfusion injury; however, this anesthetic protocol has been chosen over the alternatives of pentobarbital and inhalation anesthetics, which are recognized to have cardiorespiratory depressant (Segel and Rendig, 1986) and ischemic preconditioning effects, respectively (Stowe and Kevin, 2004).

After anesthesia and as soon as the pedal reflex had disappeared, the thoracic cage was opened and hearts were excised, weighed and immediately placed in ice-cold PBS. Hearts were then perfused in a retrograde manner, via the cannulated aorta, with a modified Krebs-Henseleit bicarbonate (KHB) buffer containing: 118 mM NaCl, 4.7 mM KCl, 1.2 mM KH_2_PO_4_, 1.2 mM MgSO_4_⋅7H_2_O, 1.25 mM CaCl_2_⋅7H_2_O, 25 mM NaHCO_3_, and 11 mM glucose, at a constant pressure of 60 mmHg. Once the left atrium was cannulated, hearts were switched to working mode perfusion and a micro-tip pressure catheter (Millar, Houston, USA or Scisense, Transonic, London, Canada) was inserted into the left ventricle prior to starting the experimental protocol.

### Experimental protocol

**Figure 1:**
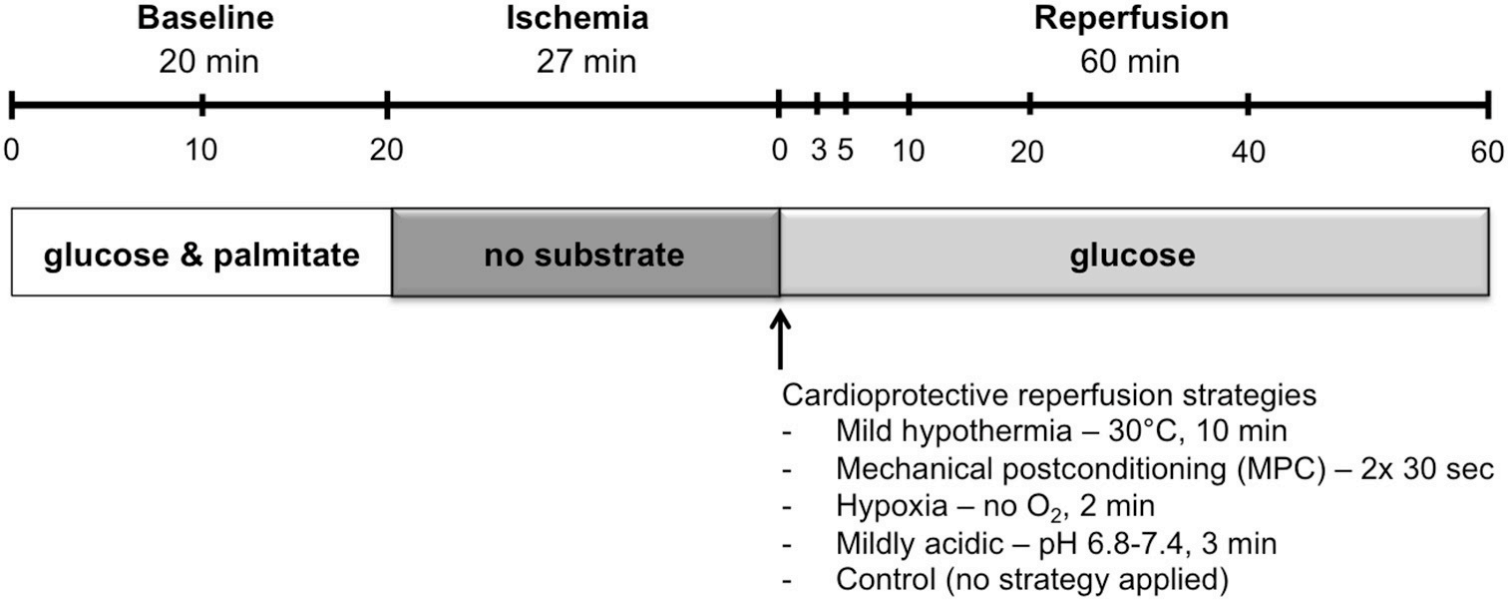
**Perfusion protocol**. Hearts underwent 20 min of baseline working-mode perfusion, followed by 27 min of warm global ischemia and 60 min of reperfusion. Hearts underwent reperfusion in the unloaded mode for the first 10 min, and were then switched to the loaded mode for the remaining 50 min. Cardioprotective reperfusion strategies, applied at the onset of reperfusion, included: mild hypothermia, mechanical postconditioning, hypoxia, and mild acid. Sample time points for preload and coronary effluent buffer are indicated in the timeline at the top of the figure.

Heart perfusion was designed to simulate DCD conditions as shown in Figure 1. A period of warm ischemia of 27 min has, in our hands, provided a reproducible and appropriate balance of post-ischemic haemodynamic recovery, permitting the evaluation of potentially cardioprotective reperfusion strategies. With shorter ischemic times, the level of recovery appeared too high to allow the quantification of a possible benefit, whereas with longer ischemic times, the damage was too severe, possibly precluding improvement. This is also in agreement with warm ischemic times reported for human DCD heart transplantations performed in Australia and the UK, which range from 11 to 24 min (Dhital et al., 2015; Page et al., 2016).

First, hearts underwent a baseline period in working mode for 20 min with KHB supplemented with 1.2 mM palmitate and 3% albumin. Hearts were excluded from the study if the mean left ventricular work (developed pressure-heart rate product) during baseline was below 23,000 mmHg^*^beats^*^ min^−1^. During working-mode baseline perfusion, afterload column height was set to 80 mmHg.

Following baseline perfusion, hearts were subjected to 27 min of global, no-flow, normothermic (37°C) ischemia: preload- and afterload-perfusate lines were clamped and the hearts were immersed in a tissue bath at 37°C containing energy-substrate–free KHB bubbled with 95% N_2_/5% CO_2_.

Reperfusion was initiated in the unloaded mode at a constant pressure of 60 mmHg for 10 min. Following unloaded reperfusion, hearts were switched to working mode for the remainder of the 60 min reperfusion. During working-mode reperfusion, mean afterload pressure was maintained at 60 mmHg with the aid of a peristaltic pump. Reperfusion was limited to 60 min as this duration is sufficient for determination of early hemodynamic recovery (Ferrera et al., 2009).

Throughout the aerobic perfusion periods, preload pressure was set at 11.5 mmHg, and perfusate buffers were maintained at 37°C and oxygenated with 95% O_2_/5% CO_2_. Samples of circulating buffer and coronary effluent were taken at several time points: at 0, 10, and 20 min during the baseline, pre-ischemic period, and at 0, 3, 5, 10, 20, 40, and 60 min during the reperfusion period (Figure 1). Circulating buffer samples were obtained immediately upstream of the heart with the aid of injection ports that allow access to perfusate lines. Coronary effluent samples were collected from the buffer that drips off the heart following its ejection from the right ventricle into the cut pulmonary artery.

Hearts from rats were randomly assigned (i.e., in an alternating fashion) before anesthesia to one of four reperfusion-strategy groups, or to the control group (no strategy applied). Controlled reperfusion strategies were applied immediately following ischemia, at the onset of reperfusion, and included: i) mild hypothermia, for which temperature of the perfusate was maintained at 30°C for 10 min followed by progressive rewarming to reach 37°C after 15 min reperfusion; ii) mechanical postconditioning (MPC), consisting of two cycles of 30 s reperfusion and 30 s ischemia, starting immediately at reperfusion; iii) hypoxia (<10% O_2_) for 2 min; and iv) acidic reperfusion at pH 6.8 with stepwise increments (0.2 pH units per min) to reach a pH of 7.4 after 3 min reperfusion.

The precise protocols used for reperfusion strategies in this study were chosen based on promising approaches reported in various models of cardiac ischemia-reperfusion injury. Although mild hypothermia during ischemia (Stadelmann et al., 2013) or for an extended period during reperfusion (Mochizuki et al., 2012) has been demonstrated to protect the heart from IR injury, its effects when applied only briefly at the time of reperfusion, have not previously been investigated. Thus, we perfused hearts at 30°C immediately upon reperfusion for 10 min. Identical MPC cycle conditions have been demonstrated to be cardioprotective using similar models of global ischemia (Ferrera et al., 2015; Bartkevics et al., 2016), and were therefore tested with the current DCD model. In addition, a small number of studies have tested hypoxic postconditioning with varying models and reperfusion conditions: either 3 min at 150 mmHg pO_2_ (Serviddio et al., 2005) or 5 min at 2% O_2_ (Angelos et al., 2006) in isolated, perfused hearts, or 5 min reoxygenation and 5 min rehypoxia in neonatal rat cardiomyocytes (Sun et al., 2005). Therefore, we decided to test the effects of no O_2_ for 2 min in our model for its clinical practicality. Several acidic reperfusion strategies have also been tested, using with ranges of pH from 6.6 to 6.8 (Inserte et al., 2008, 2011; Duan et al., 2011; Penna et al., 2013; White et al., 2016); we decided to use a mildly acidic strategy, starting at pH 6.8 and then increasing with stepwise increments of 0.2 pH units per min to reach 7.4 after 3 min, as preliminary tests with lower pH levels and rapid normalization led to worse recovery compared with controls (data not shown).

### Data collection

Intra-ventricular pressures and buffer flow (Transonic Systems Inc., Ithaca, NY, USA) in preload and afterload lines were continuously recorded using a PowerLab data acquisition system (ADInstruments, Spechbach, Germany). The following hemodynamic parameters were assessed: heart rate (beats^*^min^−1^), developed pressure (mmHg), left ventricular (LV) work (developed pressure-heart rate product; mmHg^*^beats^*^min^−1^), coronary flow (mL^*^min^−1^), cardiac output (mL^*^min^−1^), contraction and relaxation rates (dP/dt_max_ and dP/dt_min_; mmHg^*^s^−1^).

Buffer samples were used to assess metabolic parameters (oxygen consumption, lactate accumulation and cytochrome c (cyt c) release), as well as the release of necrosis markers [lactate dehydrogenase (LDH) and troponin-T (TnT)]. Oxygen consumption was determined using the Cobas b 123 blood-gas analyzer (Roche, Basel, Switzerland). Lactate accumulation was determined using a lactate assay (Sigma-Aldrich, Buchs, Switzerland) and cyt c release by an enzyme-linked immunosorbent assay (ELISA; Abcam, Cambridge, UK). LDH release was assessed using the Roche MODULAR P800 analyzer (Roche Diagnostics Corp, Indianapolis, USA), while TnT release was assessed using the Roche MODULAR E170 analyzer (Roche, Basel, Switzerland) and an electro-chemiluminescence immunoassay analyzer (Roche, Basel, Switzerland). All parameters were assessed at an early time point, 10 min reperfusion, as it may provide key information given that reperfusion injury is believed to occur in the first minutes of reperfusion, that all of our reperfusion treatments are finished at this time, and that we have previously shown that multiple parameters at this timepoint provide indications of subsequent hemodynamic recovery (Dornbierer et al., 2012; Sourdon et al., 2013).

### Data analysis

Recovery was calculated as the 60-min reperfusion time point value expressed as a percentage of the mean baseline (pre-ischemic) value.

Lactate accumulation was calculated as:

$$\begin{array}{l} \frac{\left(C_{T_{2}}*V_{T_{2}}-C_{T_{1}}*V_{T_{1}}\right)}{HW}. \end{array}$$

*C* = concentration; *HW* = heart weight; *T* = time point; *V* = buffer volume

Necrosis factor and cyt c release were calculated as:

$$\begin{array}{l} \frac{CF*\left(C_{CE}-C_{PL}\right)}{HW} \end{array}$$

*C* = concentration; *CE* = coronary effluent; *CF* = coronary flow; *HW* = heart weight; *PL* = preload

Oxygen consumption was calculated as:

$$\begin{array}{l} \frac{CF*\left(P_{PL}-P_{CE}\right)}{HW} \end{array}$$

*P* = partial pressure of O_2_; *CE* = coronary effluent; *CF* = coronary flow; *HW* = heart weight; *PL* = preload

Unless stated otherwise, values are reported as mean ± SD. Data analysis was performed with Stata (version 12.0, StataCorp, College Station, Texas). Differences between experimental groups for body weight, heart weight, hemodynamic measurements, lactate accumulation, and the release of cyt c and necrosis markers were evaluated using a linear mixed model analysis with the control group (no reperfusion strategy applied) as the reference. Mixed model analysis was also used to investigate differences between experimental groups for measurements of hemodynamic parameters and oxygen consumption at 20, 40, and 60 min reperfusion, with the corresponding baseline measurement as a covariate. We repeated the analysis for measurements at 40 and 60 min reperfusion as a sensitivity analysis, to ensure that the assumption of strict linearity for the three measurements 20, 40 and 60 min reperfusion was acceptable. For lactate release, differences between experimental groups were also analyzed with mixed models for 3–5, 3–10, and 5–10 min reperfusion, using the corresponding baseline measurement as a covariate. Model fit was assessed by inspecting the residuals. Pairwise comparisons were performed with *t*-tests for percent recovery of hemodynamic parameters and oxygen consumption. All *p*-values were two sided, adjusted for multiple comparisons (modified sequential rejective Bonferroni procedure, Holland and Copenhaver, 1987) and reported after correction. Corrected *p*-values were considered statistically significant if *p* < 0.05.

## Results

A total of 43 hearts was included in this study.

### Baseline characteristics

Rat body and heart weights, and all parameters measured during the baseline perfusion are presented in Table 1. Compared to the control group, no difference for any parameter was measured in the mild hypothermic or acidic groups. However, slight differences, compared to controls, were observed for the MPC group (lower heart rate and higher cyt c release) and as well as for the hypoxic group (higher body weight and lower LV work, heart rate, and lactate release).

**Table 1:** **Baseline characteristics**.

	**CT**	**MH**	**MPC**	**HY**	**PH**
Number of hearts (n)	10	8	9	8	8
BW (g)	387±25	405±25	395±21	414±31^‡^	400±12
HW (g)	1.95±0.18	2.05±0.17	1.96±0.27	2.00±0.26	1.90±0.15
LV work (mmHg^*^beats^*^min^−1^)	33,730±4368	33,168±1367	33,636±3571	29,689±4534^‡^	32,736±3223
HR (beats^*^min^−1^)	285±37	263±19	256±15^†^	258±16^‡^	278±18
DP (mmHg)	120±14	126±8	132±14	115±16	118±9
dP/d_tmin_ (mmHg^*^s^−1^)	−4077±713	−4085±340	−4150±567	−3963±986	−3955±604
dP/dt_max_ (mmHg^*^s^−1^)	3901±543	4033±352	4229±600	3394±666	3881±447
CO (ml^*^min^−1^)	50±5	50±5	51±8	50±7	49±8
CF (ml^*^min^−1^)	30±3	29±2	31±4	28±3	30±3
Oxygen consumption (mmHg^*^mL^*^min^−1^^*^g wet^−1^)	5174±612	4835±512	5402±761	5028±604	5401±893
Cyt c release (ng^*^min^−1^^*^g wet^−1^)	2±2	5±4	7±4^†^	3±3	3±3
Lactate accumulation (μmol^*^g wet^−1^)	9±4	9±3	7±4	4±3^‡^	7±5
LDH release (U^*^min^−1^^*^g wet^−1^)	147±171	81±123	93±108	59±62	28±39
TnT release (ng^*^min^−1^^*^g wet^−1^)	9±7	9±7	6±6	4±3	6±6

*BW, body weight; CF, coronary flow; CO, cardiac output; CT, control; cyt c release, cytochrome c release; DP, developed pressure; dP/dt_min_, minimum first derivative of LV pressure; dP/dt_max_, maximum first derivative of LV pressure; HR, heart rate; HW, heart weight; HY, hypoxia; LDH release, lactate dehydrogenase release; LV Work, left-ventricular work (HR-DP product); MH, mild hypothermia; MPC, mechanical postconditioning; PH, acidic; TnT release, Troponin T release. Data are expressed as mean ± SD*.

† MPC vs. control, p < 0.05;

‡ *HY vs. control, p < 0.05*.

### Post-ischemic recovery

As mentioned above, mixed model analyses were used to investigate differences between experimental groups for the measurements at 20, 40, and 60 min reperfusion or for only 40 and 60 min reperfusion as a sensitivity analysis (corresponding baseline measurements were included as covariates). Findings of the two analyses were almost identical, except that in the analysis with the three time points, mild hypothermia coronary flow was found to be significantly different from controls (not significant with the two time point analysis), and two significant differences with the two time point analysis were no longer significant with the three time point analysis (coronary flow for MPC vs. control and oxygen consumption for mild hypothermia vs. control). The three time-point analysis was thus considered acceptable and used for the analysis of this study.

#### Hemodynamic parameters

**Figure 2:**
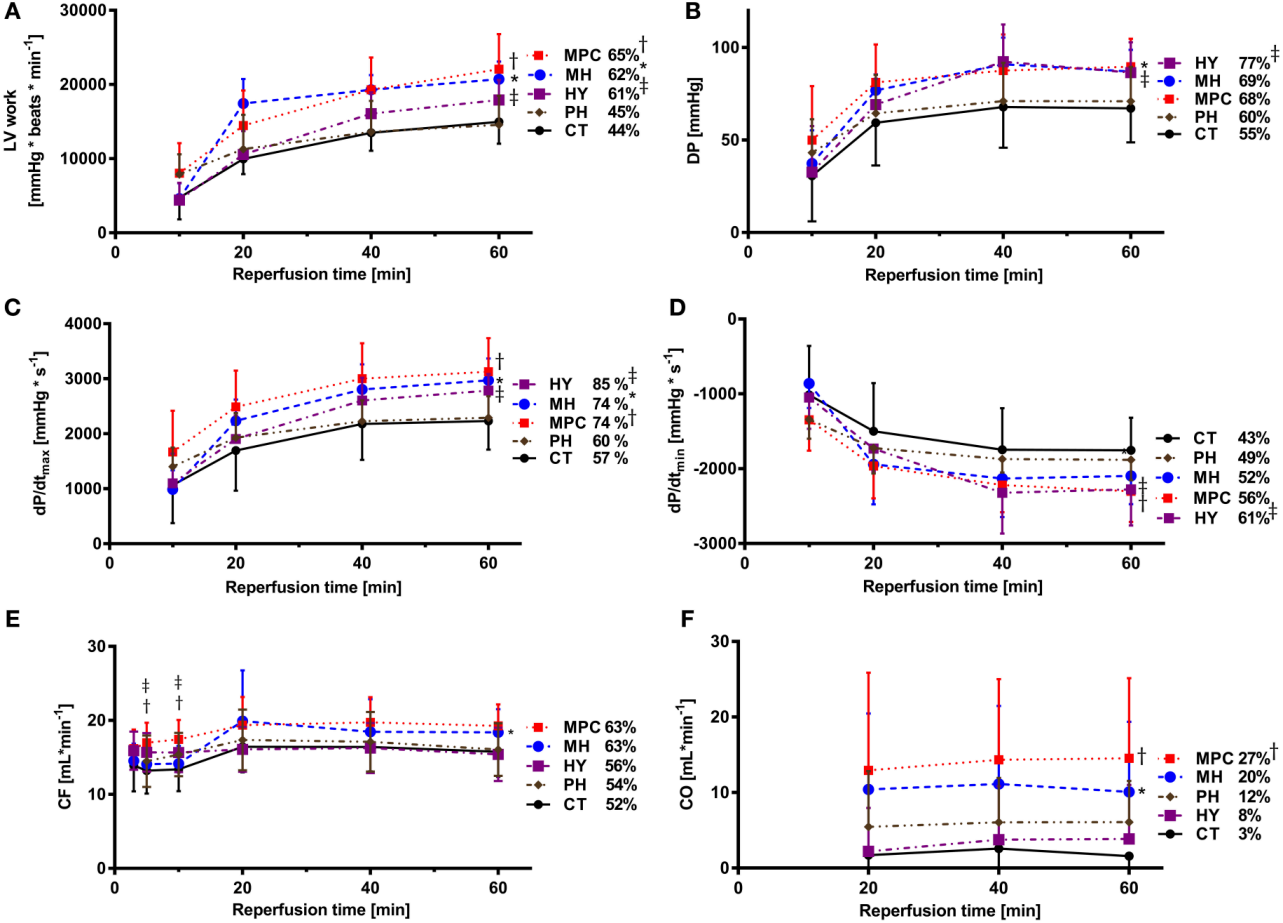
**Post-ischemic hemodynamic function. (A)** LV work (left ventricular work: HR*DP); **(B)** DP (developed pressure); **(C)** dP/dt_max_ (maximum first derivative of LV pressure); **(D)** dP/dt_min_ (minimum first derivative of LV pressure); **(E)** CO (cardiac output); **(F**): CF (coronary flow). CT, control; HY, hypoxia; MH, mild hypothermia; MPC, mechanical postconditioning; PH, acidic. Percentage of recovery corresponds to the value at 60 min reperfusion expressed as a percentage of the mean pre-ischemic values. Data are expressed as mean ± SD. ^*^MH vs. control, *p* < 0.05; ^†^MPC vs. control, *p* < 0.05; ^‡^HY vs. control, *p* < 0.05. *n* = (8–10)

Hemodynamic parameters measured during reperfusion are presented as both absolute values and percentages of pre-ischemic values in Figure 2. Post-ischemic hemodynamic recovery was improved with mild hypothermia, MPC and hypoxia, but not different with acidic reperfusion, compared to controls (Figure 2). More specifically, LV work and contraction rates (dP/dt_max_) were significantly higher with mild hypothermia, MPC and hypoxia (*p* < 0.05), while significantly higher developed pressure was measured for mild hypothermia and hypoxia (*p* < 0.05). Furthermore, relaxation rates (dP/dt_min_) were significantly higher with MPC and hypoxia (*p* < 0.05). Cardiac output was significantly higher for mild hypothermia and MPC (*p* < 0.05) compared to controls. In addition, coronary flow was significantly higher at the beginning (i.e., 5 and 10 min) of reperfusion in the MPC and hypoxia groups compared with controls (*p* < 0.05), and at the end of reperfusion for mild hypothermia vs. control (*p* < 0.05). Finally, significantly higher heart rates were measured with mild hypothermia and MPC compared to controls (data not shown).

#### Metabolic parameters

**Figure 3:**
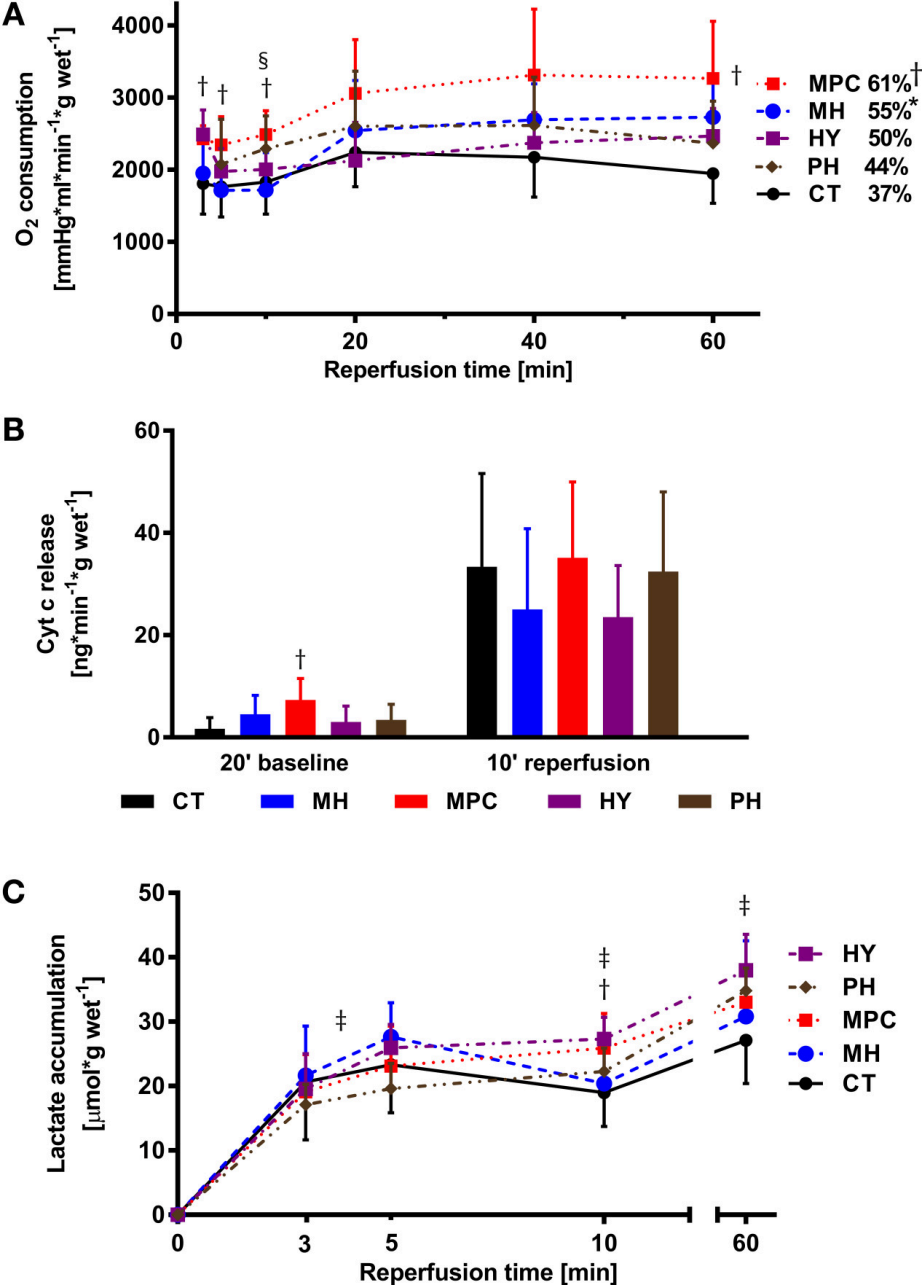
**Metabolic parameters. (A)**, Oxygen (O_2_) consumption; **(B)**, Cytochrome c release; **(C)**, Lactate accumulation. CT, control; HY, hypoxia; MH, mild hypothermia; MPC, mechanical postconditioning; PH, acidic. Percentage of recovery corresponds to the value at 60 min reperfusion expressed as a percentage of the mean pre-ischemic value. Data are expressed as mean ± SD. ^*^MH vs. control, *p* < 0.05; ^†^MPC vs. control, *p* < 0.05; ^‡^HY vs. control, *p* < 0.05; ^§^PH vs. control, *p* < 0.05. *n* = (5–10)

Significantly higher oxygen consumption was measured at the beginning of reperfusion with MPC (3, 5, and 10 min) and with acidic (10 min) groups compared to controls (*p* < 0.05), and at the end of reperfusion with mild hypothermia and MPC groups vs. controls (Figure 3A).

Cyt c release, a marker of mitochondrial integrity, tended to be lower in mild hypothermia and HY groups at 10 min reperfusion compared to control (*p* = NS; Figure 3B).

During reperfusion, we measured higher lactate accumulation between 3 and 5 min reperfusion in hypoxia vs. control (*p* < 0.05; Figure 3C), at 10 min in MPC and hypoxia, and at 60 min in hypoxia compared to control (*p* < 0.05 for all).

#### Markers of cell death

**Figure 4:**
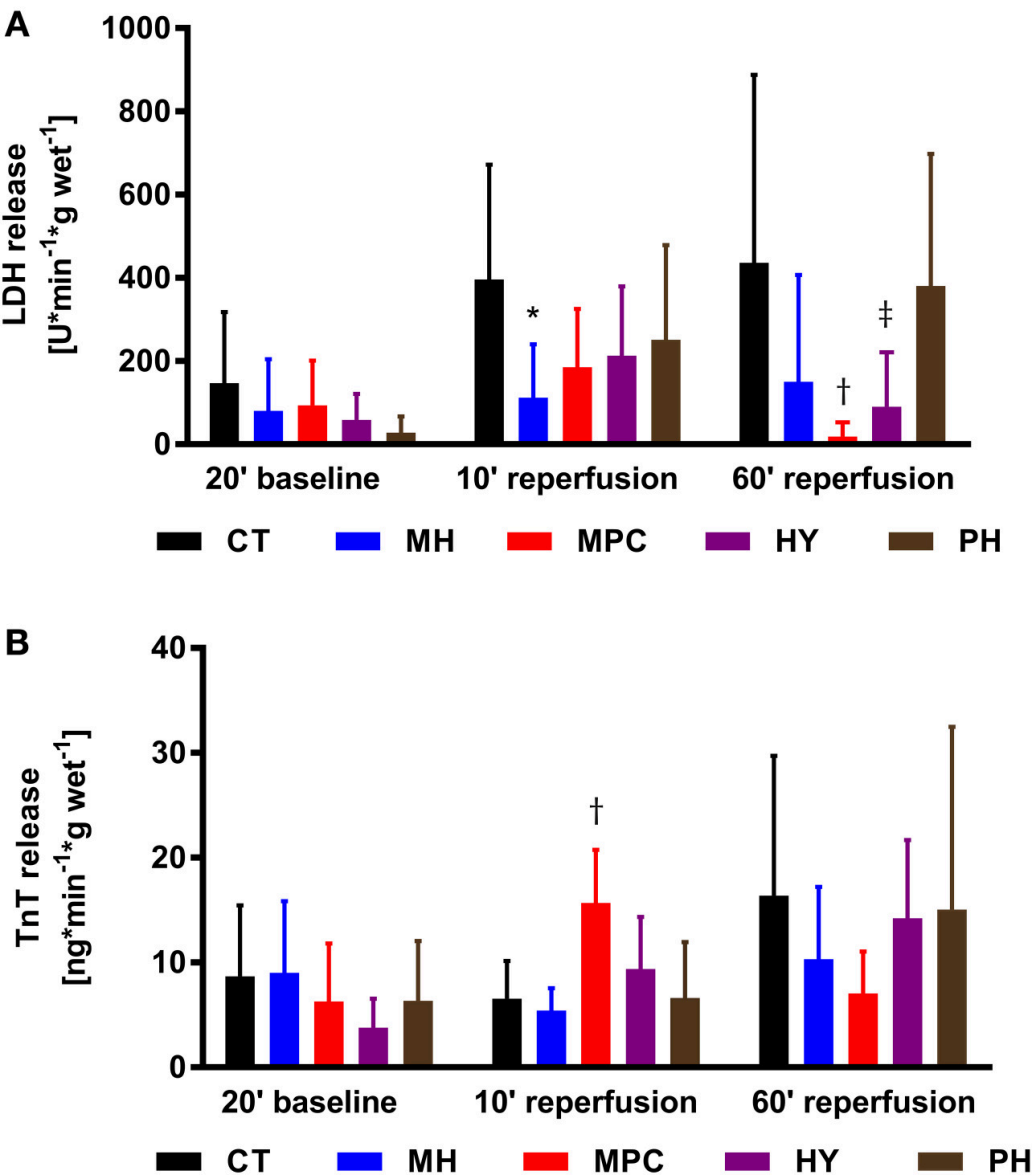
**Necrosis markers. (A)**, LDH (lactate dehydrogenase) release; **(B)**, TnT (Troponin T) release. CT, control; HY, hypoxia; MH, mild hypothermia; MPC, mechanical postconditioning; PH, acidic. Data are expressed as mean ± SD. ^*^MH vs. control, *p* < 0.05; ^†^MPC vs. control, *p* < 0.05; ^‡^HY vs. control, *p* < 0.05. *n* = (6–10)

LDH and TnT were measured as markers of cell death (Figure 4). LDH release was significantly decreased at 10 min reperfusion for mild hypothermia and at 60 min reperfusion for MPC and hypoxia compared to controls (*p* < 0.05 for all). We also measured a higher TnT release at 10 min reperfusion in the MPC group vs. controls (*p* < 0.05).

## Discussion

We report that short, controlled reperfusion strategies, applied immediately following ischemia, effectively improve cardiac hemodynamic recovery following global, normothermic ischemia in our isolated working rat heart model of DCD. Hearts subjected to mild hypothermia, mechanical postconditioning (MPC), or hypoxia at reperfusion showed a significantly higher post-ischemic hemodynamic recovery compared to controls (i.e., no strategy applied), but acidic reperfusion provided no benefit. Assessment of metabolic and necrosis parameters support the concept that differing, and potentially complementary, mechanisms underlie the improvements associated with these early reperfusion cardioprotective strategies.

To our knowledge, this is the first report that mild hypothermia (30°C), when applied only at the *onset* of reperfusion for a *brief* period (10 min), improves post-ischemic heart function. We demonstrate that this reperfusion strategy significantly improved recovery of LV work, developed pressure, heart rate, contraction rate and cardiac output compared with controls. This was associated with reduced LDH release, indicating less necrosis, and higher coronary flow, suggesting improved endothelial function, as well as increased oxygen consumption and a tendency for less cyt c release, which together imply a better metabolic recovery. It has previously been reported that mild hypothermia can improve heart recovery when applied during ischemia (Stadelmann et al., 2013), during ischemia and reperfusion (Shao et al., 2007, 2013), or when applied during reperfusion if prolonged to 60 (Tolboom et al., 2016) or 180 (Mochizuki et al., 2012) min. Pro-survival kinases [protein kinase C, protein kinase B (Akt)], endothelial nitric oxide synthase (eNOS) and nitric oxide (NO), in addition to energy preservation, have been reported to underlie some benefits provided by mild hypothermia (Tissier et al., 2012). Although intracellular signaling mechanisms were not explored in the current study, our findings of greater post-ischemic coronary flow and potentially improved endothelial function are consistent with increased Akt and eNOS activities and elevated NO levels reported previously (Shao et al., 2007; Mochizuki et al., 2012).

We also demonstrate that MPC is effective in improving post-ischemic recovery of several parameters. Hemodynamic parameters, including left ventricular work, heart rate, contraction rate, relaxation rate, coronary flow and cardiac output, were significantly greater than those for control hearts during reperfusion, which is consistent with previous reports in similar, working (loaded) heart models (Lauzier et al., 2007; Bartkevics et al., 2016). Interestingly, MPC may also promote glucose metabolism (Correa et al., 2008) and its protective effects appear to depend on energy substrate availability (Bartkevics et al., 2016). More specifically, we previously found that the effect of MPC was dependent on the circulating energy substrate levels at reperfusion; MPC improved post-ischemic hemodynamic recovery in hearts reperfused with fatty acids and glucose, but not in hearts reperfused with glucose only (Bartkevics et al., 2016). This effect is consistent with the requirement for MPC-induced increases in glucose metabolism for its cardioprotective effects (Correa et al., 2008); as in hearts reperfused with glucose only, glucose metabolism may already be maximal, and can therefore not be increased by MPC. Interestingly, in the present study, MPC also improved hemodynamic recovery in hearts exposed to high levels of circulating fatty acids, but reperfused with glucose only. Thus, it is possible that in the current study, MPC promoted improved hemodynamic recovery by increasing glucose metabolism, and thereby inhibiting oxidation of residual fatty acids during early reperfusion. Indeed, stimulation of glucose metabolism and inhibition of fatty acids during early reperfusion is associated with better hemodynamic recovery (Lopaschuk et al., 2003; Ussher et al., 2012). Importantly, the effects of energy substrate metabolism on MPC may contribute to variable cardioprotective benefits in clinical trials (Hausenloy and Yellon, 2016) and have not been thoroughly investigated. We are currently evaluating metabolic changes induced by MPC to further characterize this concept. Importantly, Ferrera et al. (2015) recently reported improvements in hemodynamic recovery with MPC and low-pressure reperfusion in an unloaded isolated heart model. Of particular interest, low-pressure reperfusion resulted in higher post-ischemic recovery compared to MPC when it was applied after 3 min of reperfusion. Thus, low-pressure reperfusion represents an additional, promising strategy to improve post-ischemic cardiac recovery in DCD hearts.

Cardioprotection provided by MPC is believed to be mediated by a combination of passive effects (slowing the normalization of intracellular homeostasis) and active effects (recruitment of pro-survival intracellular signaling pathways [reperfusion injury salvage kinase (RISK) and survivor activating factor enhancement (SAFE) (Tsang et al., 2004; Bopassa et al., 2006; Lacerda et al., 2009)], opening of mitochondrial ATP-dependent potassium channel (Garlid et al., 2009), and inhibition of mPTP opening (Hausenloy et al., 2002; Argaud et al., 2005). Given that MPC has previously been demonstrated to improve mitochondrial function (Bopassa et al., 2005, 2006), one might expect lower cyt c release at reperfusion following MPC. However, cyt c release at reperfusion was not different with MPC compared with controls, while it was significantly greater at baseline. It is possible that a lower cyt c release could have been measured at reperfusion, if no differences in cyt c release had been measured at baseline. In contrast, we demonstrate that MPC significantly improved oxygen consumption compared with controls, implying a better metabolic recovery, as would be expected with preserved mitochondrial integrity. In addition, we demonstrate that MPC induced a greater coronary flow during reperfusion compared with controls suggesting improved endothelial function.

In our model, brief hypoxic reperfusion performed immediately after ischemia improved hemodynamic function (LV work, developed pressure, contraction rate, relaxation rate, and coronary flow) compared with oxygenated reperfusion (controls). This is in agreement with Serviddio et al. (2005) who reported that the LV developed pressure was improved after a short period of hypoxia at the onset of reperfusion, although this study was performed under different and potentially less severe ischemic conditions; hearts were cooled (4°C) and protected with a cardioplegic solution. A brief period of hypoxia at the onset of reperfusion should benefit the heart by delaying the reoxygenation of the ischemic myocardium and thereby reducing reactive oxygen species production and the damaging effects of oxidative stress, which include disruption of mitochondrial integrity (Serviddio et al., 2005; Yellon and Hausenloy, 2007; Thu et al., 2010). Correspondingly, we observed a tendency toward reduced cyt c release at reperfusion, suggesting less mitochondrial damage compared to controls. We also measured higher coronary flow at the beginning of reperfusion, suggesting improved endothelial function. Further, several studies have also demonstrated beneficial effects of a brief period of hypoxic reperfusion after ischemia (Serviddio et al., 2005; Sun et al., 2005; Angelos et al., 2006). These studies were performed using either Langendorff-perfused hearts or cells, and therefore, lack information concerning the pump function of the heart. Moreover, these experimental models were not specifically designed for DCD, either having a cold ischemia and/or not using clinically relevant energy substrates (high levels of circulating fatty acids in the pre-ischemic period).

We also evaluated the possible benefit of an initial brief period of mildly acidic reperfusion. Although it has been reported to improve hemodynamic recovery (Inserte et al., 2008, 2011; Duan et al., 2011; Penna et al., 2013; White et al., 2016), infarct size (Inserte et al., 2008; Penna et al., 2013), ATP levels (Inserte et al., 2008), and to decrease cyt c release and mitochondrial swelling (Duan et al., 2011) and markers of cell death (Inserte et al., 2008; Duan et al., 2011), we did not observe hemodynamic benefits, nor did we see a difference, compared to controls, in necrosis parameters. Although we measured higher oxygen consumption at 10 min reperfusion, we did not see other differences in metabolic recovery. Mildly acid reperfusion is believed to act by delaying the restoration of physiological pH (Inserte et al., 2008, 2011), thereby prolonging the low-pH–induced inhibition of mPTP opening while reducing intracellular calcium overload and hypercontracture in the first minutes of reperfusion. Discrepant cardioprotective effects for mildly acidic reperfusion between previous studies (Inserte et al., 2008; Duan et al., 2011; Inserte et al., 2011; Penna et al., 2013; White et al., 2016) and ours are unlikely to be explained by differences in the acidic reperfusion conditions (previous studies used pH ranging from 6.4 to 6.8 for 2–3 min, while we used pH 6.8 for 3 min), but may be explained by differences in experimental conditions, as several previous studies used longer ischemic periods [30–40 min vs. 27 min in our study; (Inserte et al., 2008, 2011; Duan et al., 2011; Penna et al., 2013)] and/or higher buffer calcium concentrations 1.4–1.8 mM (Inserte et al., 2008, 2011; Duan et al., 2011) or lower (0.22 mmol/L) (White et al., 2016) vs. 1.25 mM in our study. Taken together, conditions in several previous reports are likely to generate a more severe ischemia than in our study, which may lead to conditions that are more damaging, but put hearts in a position to benefit from mildly acidic reperfusion. Moreover, combined conditions such as a hypocalcemic, acidic solution (White et al., 2016) might be more effective in improving post-ischemic recovery than a normocalcemic, acidic solution, as used in our study.

Markers of cell death, release of LDH and TnT into the coronary effluent, were monitored to provide insight into the mechanisms underlying the cardioprotective effects of reperfusion strategies. Similar patterns for LDH and TnT release were expected, and generally observed for baseline and 60 min reperfusion time points; at 60 min reperfusion LDH and TnT tended to be lower for MPC and hypoxia compared to controls, suggesting less cell death with these two strategies. However, TnT, but not LDH, release at 10 min reperfusion was significantly higher in the MPC group vs. controls (*p* < 0.05). The reason for this discrepancy is not immediately obvious. It is worth noting that assessments of LDH and TnT were performed at 10 and 60 min reperfusion; early time points compared with clinical measurements which are typically performed at hours or days after an ischemic insult. Nonetheless, useful information can be gained from these early time points; we have previously demonstrated that release of LDH, but not TnT, at 10 min reperfusion is an early predictive parameter of subsequent hemodynamic recovery in an isolated rat heart model of DCD (Sourdon et al., 2013). Therefore, the timing of release of LDH and TnT is likely not identical during early reperfusion, and LDH at 10 min reperfusion may provide a better indicator of recovery than TnT.

### Limitations

Our findings support the concept that controlled reperfusion strategies may be useful to optimize post-ischemic function in heart grafts obtained with DCD; nonetheless, several limitations exist. First, the anesthetics used in this study are ketamine and xylazine, which have been shown to interfere with the sympathetic nervous system (Juang et al., 1980; Svorc et al., 2013) and could have a possible confounding effect on ischemia reperfusion injury. Nonetheless, we expect to have similar effects of the anesthetics in all the experimental groups. In addition, these results were obtained in an isolated rat heart model and are as such not fully representative of a clinical scenario. Indeed, extrapolation of results obtained in animal experiments to clinical scenarios certainly depends on the species being evaluated (Rossello and Yellon, 2016) and differences in animal physiology, including species-specific sensitivity to myocardial ischemia reperfusion (Lecour et al., 2014). As a consequence, additional preclinical testing is required, including evaluations in a larger animal model. Finally, maintenance of post-ischemic benefits obtained with these strategies applied at the onset of reperfusion must be confirmed following storage and transplantation.

### Conclusion

In the current study, we demonstrate that improving the heart function after normothermic, global ischemia is possible through various controlled reperfusion strategies including mild hypothermia, mechanical postconditioning and hypoxia. Differing underlying mechanisms seem to be involved. Thus, a combination of these strategies might be necessary to achieve further improvement in post-ischemic function and may also provide a more robust strategy that could be of use in the establishment of clinical protocols for DCD heart transplantation.

## Author contributions

Conception and research design: EF, PN, HS, SL. Experimental work and data analysis: EF, PN, RW, NM, BG, GF, SL. drafting, editing, revision and approval of final version of manuscript: EF, PN, RW, NM, BG, GF, TC, HT, SL.

## Funding

This work was supported by a Project Grant from the Swiss National Science Foundation (310030_149730/1) and a Research Grant from the Ruth & Arthur Scherbarth Foundation. The funders had no role in study design, data collection and analysis, decision to publish, nor in preparation of the manuscript.

### Conflict of interest statement

The authors declare that the research was conducted in the absence of any commercial or financial relationships that could be construed as a potential conflict of interest.
